# PTEN loss regulates alveolar epithelial cell senescence in pulmonary fibrosis depending on Akt activation

**DOI:** 10.18632/aging.102262

**Published:** 2019-09-17

**Authors:** Ting Qiu, Yaqiong Tian, Yujuan Gao, Miao Ma, Hui Li, Xiaoqin Liu, Hongyan Wu, Yingwei Zhang, Hui Ding, Mengshu Cao, Ji Zhang, Jinghong Dai, Jingyu Chen, Hourong Cai

**Affiliations:** 1 Department of Respiratory Medicine, Drum Tower Clinical Medical College of Nanjing Medical University, Nanjing 210008, Jiangsu, People’s Republic of China; 2 Department of Respiratory Medicine, The Affiliated Drum Tower Hospital of Nanjing University Medical School, Nanjing 210008, Jiangsu, People’s Republic of China; 3 Department of Respiratory Medicine, KunShan Hospital of Traditional Chinese Medicine, Kunshan, Jiangsu 215300, People’s Republic of China; 4 Department of Pathology, The Affiliated Drum Tower Hospital of Nanjing University Medical School, Nanjing 210008, Jiangsu, People’s Republic of China; 5 Department of Respiratory Medicine, The Affiliated Yixing People Hospital, Jiangsu University, Yixing 214200, Jiangsu, People’s Republic of China; 6 Jiangsu Key Laboratory of Organ Transplantation, Wuxi People’s Hospital, Nanjing Medical University, Wuxi 214023, Jiangsu, People’s Republic of China

**Keywords:** aging, cellular senescence, pulmonary fibrosis, phosphatase and tension homolog deleted on chromosome ten, protein kinase B

## Abstract

Idiopathic pulmonary fibrosis (IPF) is an aging-associated disease with poor prognosis. The mechanisms underlying the role of alveolar epithelial cell (AEC) senescence in IPF remain poorly understood. We aimed to investigate if PTEN/Akt activates AEC senescence to induce pulmonary fibrosis. We investigated the association between PTEN/Akt and cellular senescence in lung tissues from IPF patients. As a result, decreased PTEN and activated Akt pathway were found in AECs in fibrotic lung tissues detected by immunohistochemistry (IHC) and immunofluorescence (IF). Increased expression levels of aging-associated markers (P21^WAF1^ and SA-β-gal) in AECs treated with bleomycin were found. AEC senescence was accelerated by PTEN knockdown and attenuated by PTEN overexpression. Bleomycin induced AEC senescence was reversed by Akt2 knockdown and the pharmacological inhibitors (LY294002 and MK2206) of the Akt pathway. Reducing Akt activation dramatically improved lung fibrosis in a fibrotic mice model. In addition, a co-immunoprecipitation (co-IP) assay demonstrated that PTEN physically associated with Akt. These indicated that senescent AECs modulated by the PTEN/Akt pathway promote lung fibrosis. In conclusion, our study demonstrated that as a trigger indicator in IPF, the senescence process in AECs should be a potential therapeutic target and that the PTEN/Akt pathway may be a promising candidate for intervention.

## INTRODUCTION

Idiopathic pulmonary fibrosis (IPF) is a specific form of chronic, progressive, fibrosing interstitial pneumonia [1] with a median survival rate of 3.8 years after diagnosis [2]. IPF is characterized by excessive scar tissue formation and destruction of lung parenchyma, resulting in gas exchange abnormalities and respiratory failure. The annual cumulative prevalence increased steadily from 202.2 cases per 100000 people in 2001 to 494.5 cases per 100000 people in 2011 in the USA. Currently, drug treatment cannot definitively improve the quality of life for IPF patients or “reverse” fibrosis.

The incidence of IPF increases with age, and it is now widely regarded as a disease of aging [3]. IPF patients under 50 years of age are rare [4]. Two-thirds of IPF patients are older than 60 years at the time of presentation with a mean age of 66 years at the time of diagnosis. Furthermore, the survival rate of IPF patients markedly decreases with age [5]. Aging is considered the main risk factor for IPF [6]. Different mechanisms associated with aging, including stem cell dysfunction [7], aging mediator overexpression [8], and mitochondrial superoxide [9], have been described to participate in the pathogenesis of IPF.

Increasing evidence has implicated that defects in alveolar epithelial cells (AECs) induce lung fibrosis. Histopathologic abnormalities of AEC have been observed in tissue sections from IPF patients and in animal models of pulmonary fibrosis [10]. Mutations in AEC genes, including surfactant proteins A and C, have been linked to familial disease [11]. Transgenic animal experiments have confirmed that targeted injury to AEC is sufficient to initiate lung fibrosis [12, 13].

Mechanisms of AEC injury and dysregulated repair responses have been proposed to be critically involved in the progression of IPF [14]. Senescence of AEC may be detrimental to lung repair. Depletion of senescent epithelial cells in vitro and ex vivo decreases fibrotic markers [15]. However, the pathogenesis of AEC senescence involved in IPF is largely unknown.

Phosphatase and tension homolog deleted on chromosome ten (PTEN) has recently drawn extensive attention for its critical anti-tumor function, as it regulates several fundamental cellular processes, including cell adhesion, growth, migration and apoptosis [16]. Several studies have investigated the effects of PTEN on longevity [17, 18]. Interestingly, while monoallelic loss or mutation of PTEN gene drives cellular proliferation, complete inactivation of PTEN induces a senescence response in tumor cells [19]. Additionally, previous studies have investigated the role of PTEN in aging-related renal impairment [20]. Genetic ablation of PTEN in prostatic epithelial cells induces cell senescence [21]. Thus, we posited that PTEN may be involved in the pathogenesis of the senescence-associated molecular mechanism of IPF.

The protein kinase B (Akt) signaling pathway, which is negatively regulated by PTEN, plays a critical role in the regulation of cell growth and cell survival in various systems. The Akt pathway also constitutes an endogenous negative feedback regulator that reduces oxidative stress and limits pro-inflammatory and apoptotic events in response to injury [22]. There have been many attempts to investigate the involvement of the Akt pathway in the aging process [23–26]. It remains poorly understood if PTEN-regulation of the Akt pathway during AEC senescence is involved in the pathogenesis of pulmonary fibrosis.

In this study, we confirmed that AECs possess senescent properties in IPF patients. Bleomycin-stimulated AECs in vitro and a bleomycin-induced mouse pulmonary fibrosis model demonstrated that the loss of PTEN causes senescence in AECs through activation of the Akt pathway. Knockdown of Akt or inhibition of Akt activation reverses this change.

## RESULTS

### IPF is a disease of aging

**Figure 1:**
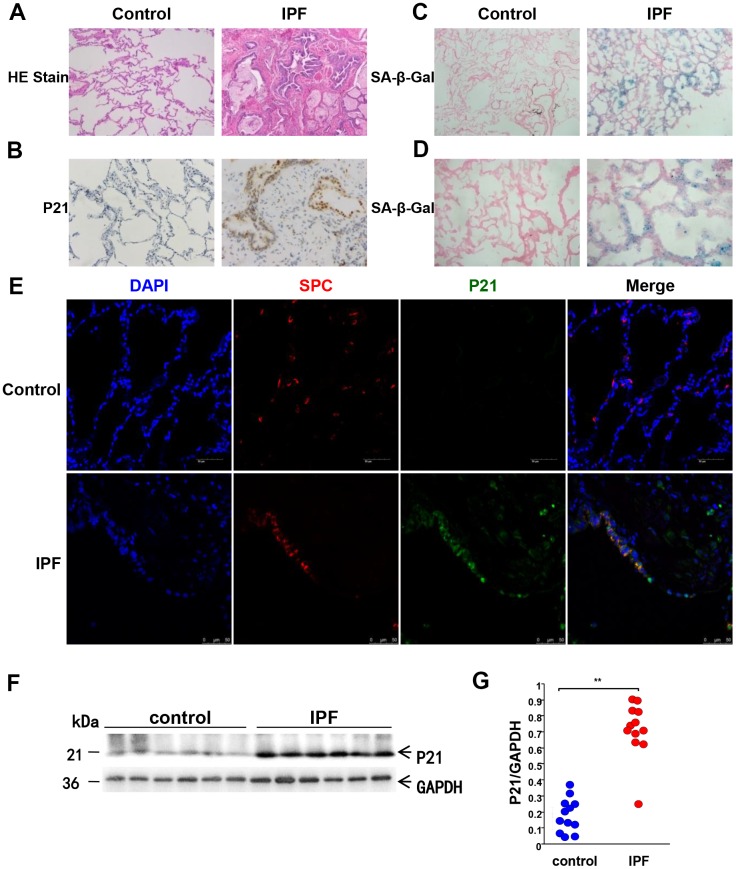
**Aging-related markers are significantly overexpressed in the lung tissues of IPF patients.** Human lung tissues of IPF (n = 12) and age-matched normal lung tissues (n = 12) were analyzed. (**A**) Representative images of HE staining of lung tissues (original magnification, 100×). (**B**) Representative images of the aging-related marker, P21^WAF1^, via IHC staining of lung tissues (original magnification, 100× in control and 200× in IPF). (**C**) Representative results of SA-β-Gal staining of lung tissues (original magnification, 100×). (**D**) Enlarged images of SA-β-Gal staining (200×). (**E**) Immunofluorescence staining of SP-C (an AEC2-specific marker, red) and P21^WAF1^(green) was conducted to confirm senescent marker expression in AECs (original magnification, 400×). (**F**, **G**) P21^WAF1^ senescent marker in lung tissues was measured by western blotting. Each dot represents an individual lung tissue. **p < 0.01. Unpaired, two-tailed Student’s t test.

In this study, we used the aging-related markers, P21^WAF1^ and SA-β-Gal, to detect senescence in IPF lung tissues and AECs. P21^WAF1^ was detected by methods including western blotting, IHC and immunofluorescence staining. As shown in Figure 1, both senescent markers, P21^WAF1^ and SA-β-Gal, were over-expressed in the lung tissues from IPF patients compared to age-matched normal controls (Figure 1A–1G). HE and IHC staining showed a destroyed alveolar structure (Figure 1A, 1B), and the positive staining of P21^WAF1^ was primarily located in AECs of fibrotic areas in the lung tissues of IPF patients. In addition, strongly positive SA-β-Gal staining was primarily located in AECs from lung tissues with IPF, whereas SA-β-Gal staining was barely observed in normal lung tissues (Figure 1C, 1D). Furthermore, immunofluorescence was used to examine the spatial location of P21^WAF1^ and AEC2 markers (SP-C), in IPF lung and normal tissues (Figure 1E). P21^WAF1^ was overexpressed in IPF lung tissue but was barely present in normal lung tissue. Western blotting confirmed P21^WAF1^ expression differences between IPF and normal lung tissues (Figure 1F, 1G). Taken together, these data suggested that IPF is an aging-related disease as previous findings demonstrated and abnormal senescence occurs in AECs from IPF lung tissues.

### PTEN loss and Akt pathway activation in AECs from IPF

**Figure 2:**
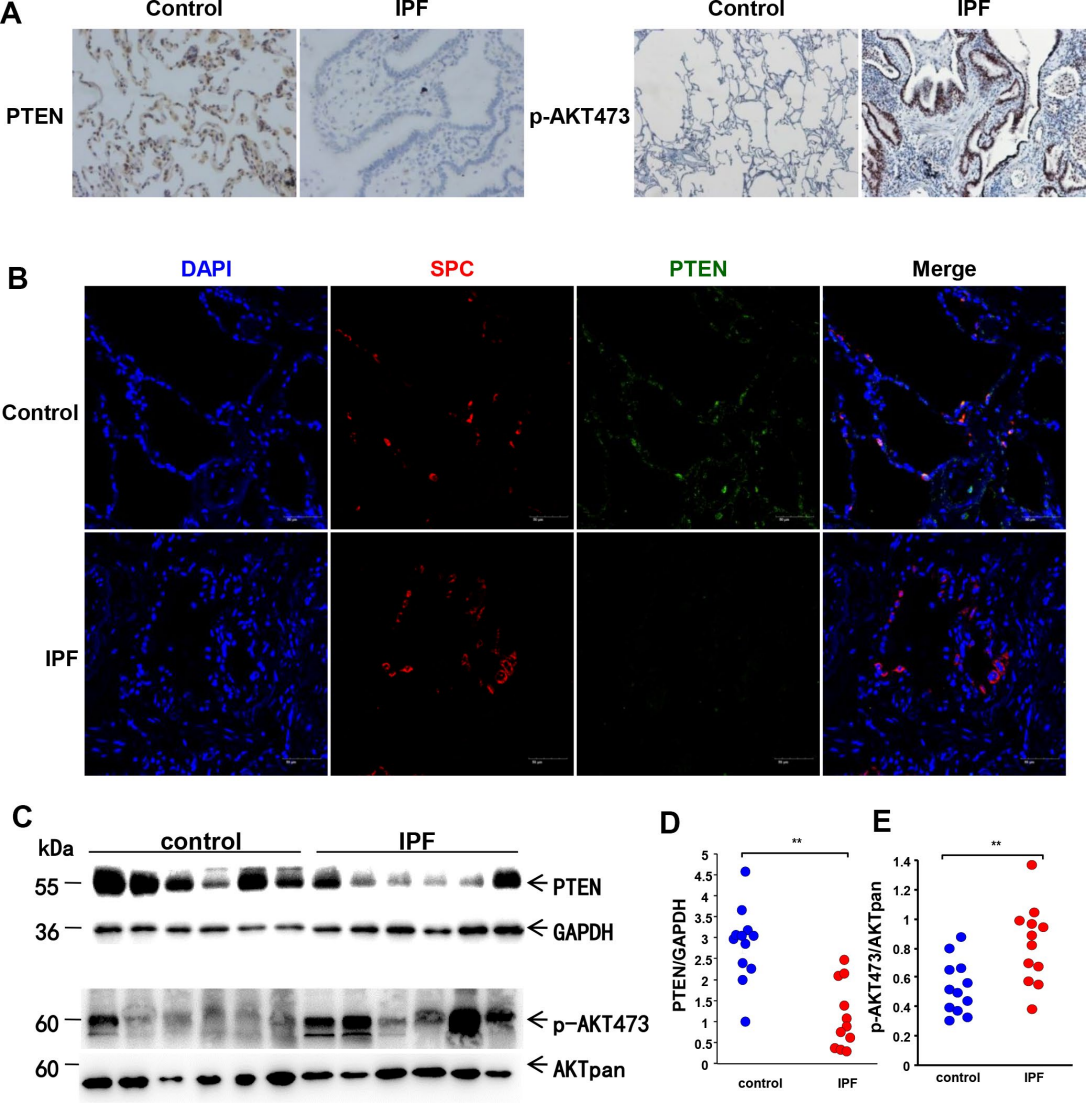
**Loss of PTEN and activation of the AKT pathway in lung tissue from IPF patients.** (**A**) Representative results of IHC staining for PTEN and p-AKT 473 in lung tissues (original magnification, 200× for PTEN and 100× for p-AKT 473). (**B**) Immunofluorescence staining for both SP-C (red) and PTEN (green) were conducted to examine the spatial distribution of PTEN (original magnification, 200×). (**C**–**E**) Western blot analysis was applied to detect the expression of PTEN and activation of the AKT pathway. Each dot represents an individual lung tissue. **p < 0.01. Unpaired, two-tailed Student’s t test.

After demonstrating that aging-related markers were overexpressed in AECs from IPF lung tissues, we next investigated if PTEN and the Akt pathway participated in senescence of AECs from IPF lung tissues. As indicated in Figure 2A, the phosphorylation levels of Akt were higher in IPF lung tissues than in normal lung tissues, while the level of PTEN was lower in IPF. Both markers were observed predominantly distributed within AECs in fibrotic areas. western blot analysis was then performed using IPF lung tissues and normal lung tissues. The results of PTEN and p-Akt 473 were consistent with IHC staining (Figure 2C–2E). To further confirm PTEN location, we conducted immunofluorescence staining. As shown in Figure 2B, PTEN was downregulated in IPF lung tissues. In normal lung tissues, PTEN was mainly distributed in AECs, but some PTEN colocalized with SP-C. Therefore, these data suggested that the decreased expression of PTEN may be related to Akt activation in IPF and that this regulation primarily occurs within AECs. To further confirm our findings from clinical samples, in vitro experiments were performed to elucidate the mechanisms underlying AEC senescence.

### PTEN is decreased in bleomycin-induced AEC senescence and Akt pathway phosphorylation levels are increased

**Figure 3:**
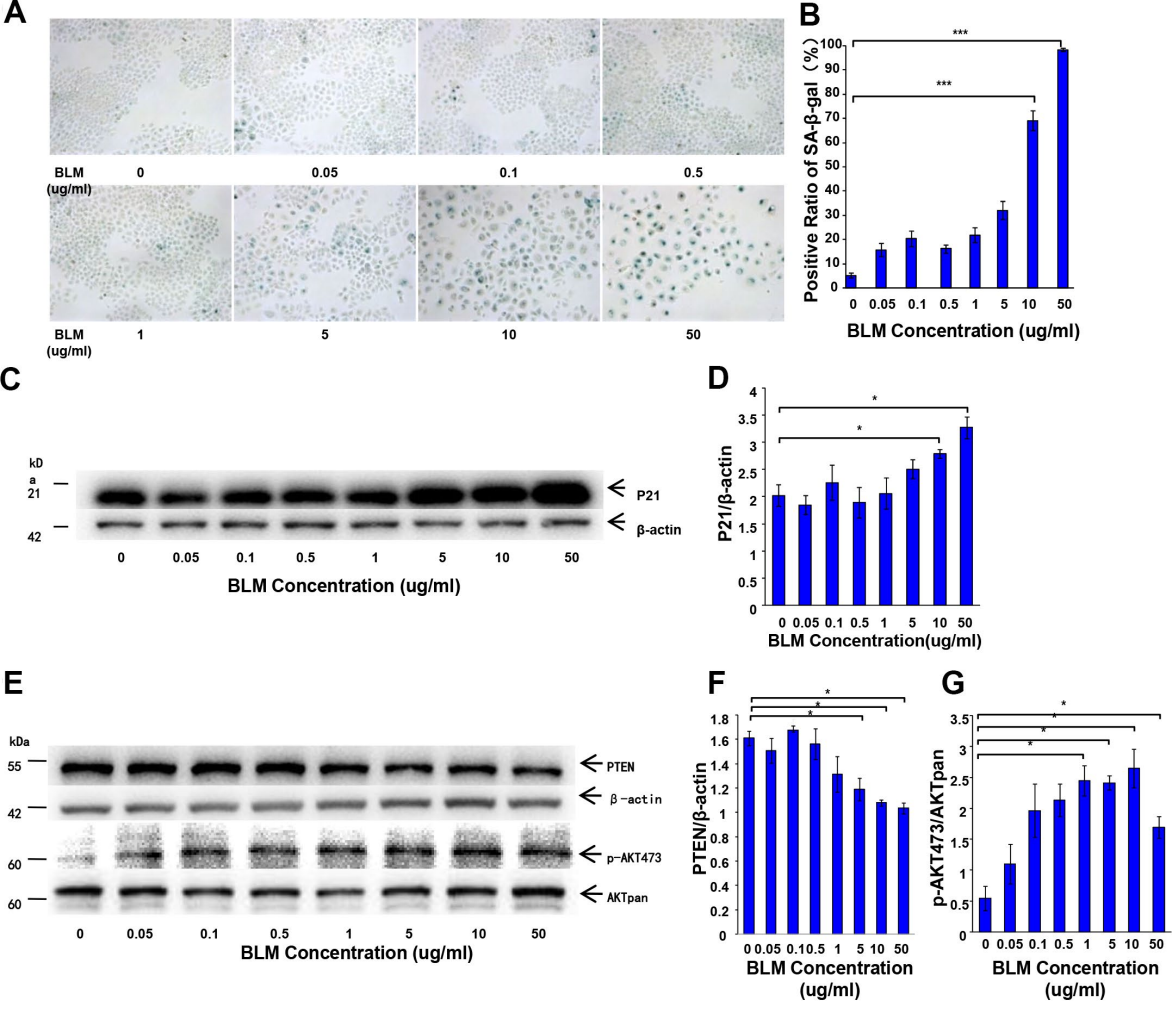
**Decrease of PTEN and activation of the AKT pathway in the senescent cell model.** Gradually increasing concentrations of bleomycin was added to culture medium to stimulate A549 cells for 72 hours followed by transfer to fresh FBS-free medium for another 24 hours. (**A**, **B**) SA-β-Gal staining was performed to detect cellular senescence (original magnification, 200×). (**C**, **D**) The expression of the aging-related marker, P21^WAF1^, was detected by western blot analysis. (**E**–**G**) PTEN loss and AKT pathway activation were observed. Data are shown as the mean ± SEM, n ≥ 3 per group. *p < 0.05, ***p < 0.001. One-way ANOVA followed by Dunnett’s Multiple Comparison Test.

Bleomycin has been used to induce AEC senescence in previous studies. Here, we employed bleomycin to generate a cellular senescence model using A549 cell lines. In many studies, A549 is generally used as a replacement for primary AECs because AECs are difficult to obtain and maintain in culture ex vivo. Gradually increasing concentrations of bleomycin were added to culture medium to stimulate A549 cells for 72 hours, and cells were then transferred to fresh FBS-free medium for another 24 hours to generate the cellular senescence model. SA-β-Gal staining and western blotting were performed. As shown in Figure 3, the intensity of positive SA-β-Gal staining increased with increasing concentrations of bleomycin (Figure 3A, 3B). In addition, the senescence-related marker, P21^WAF1^, was overexpressed in a dose-dependent manner in stimulated A549 cells (Figure 3C, 3D). To investigate the role of PTEN and the Akt pathway during bleomycin-induced cellular senescence, we examined the expression of PTEN and Akt in bleomycin-induced senescent A549 cells. As shown in Figure 3E–3G, PTEN was significantly reduced with increasing concentrations of bleomycin, and activation of the Akt pathway was detected. These data suggested that loss of PTEN activated the Akt pathway to promote AEC senescence, thus participating in the pathogenesis of IPF. For further examination, we conducted a set of in vitro experiments to elucidate the potential mechanisms underlying cellular senescence in IPF.

### Change of PTEN expression affects AEC senescence and Akt activation

**Figure 4:**
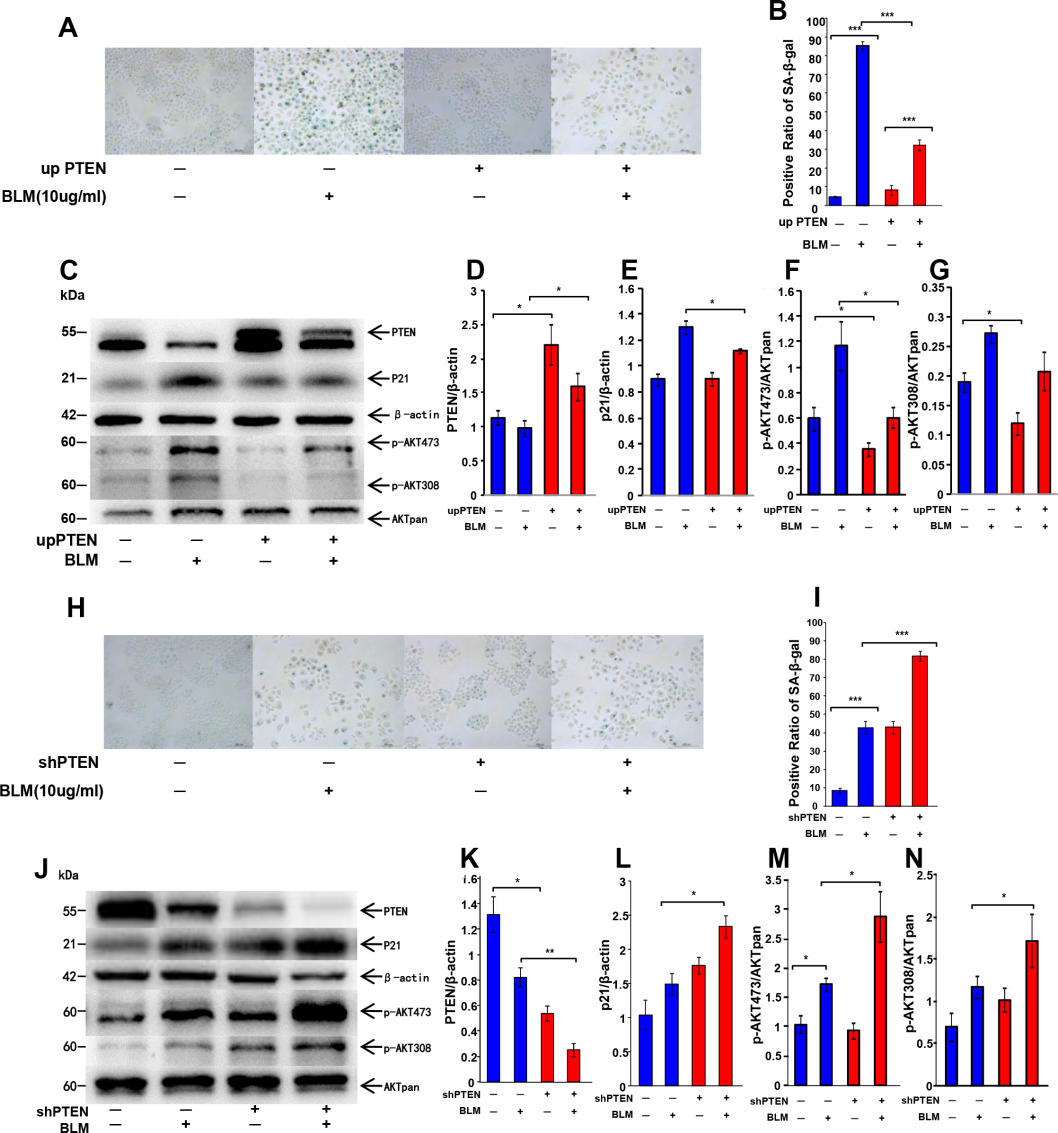
**Change of PTEN expression affects cell senescence through AKT pathway activation.** Genetic overexpression of PTEN in A549 cells was achieved by a transduction of a lentiviral vector followed by bleomycin (10 μg/ml) stimulation for 72 hours and transfer to fresh medium for 24 hours. (**A**, **B**) Cellular senescence was detected by SA-β-Gal staining (original magnification, 200×). (**C**–**G**) Western blot analysis was performed to confirm alteration of P21^WAF1^ and targets of the PTEN/AKT pathway. Lentiviral vector was used to knockdown PTEN expression in A549 cells. Bleomycin was then added to the culture medium to stimulate cells for 72 hours followed by transfer to fresh FBS-free medium for another 24 hours. (**H**, **I**) SA-β-Gal staining was applied to detect cellular senescence (original magnification, 200×). (**J**–**N**) Change of P21^WAF1^ and expression of PTEN/AKT targets were determined by western blotting. Data are shown as the mean ± SEM, n ≥ 3 per group. *p < 0.05, **p < 0.01, ***p < 0.001. One-way ANOVA followed by Dunnett’s Multiple Comparison Test.

Although our initial experiments showed reduced PTEN expression and elevated Akt activation in IPF lung tissues and in the senescence cell model, it remained uncertain if decreased PTEN and increased Akt activation were responsible for the induction of AEC senescence. To determine if the change of PTEN expression affects AEC senescence through the activation of Akt, PTEN was overexpressed or knocked down in A549 cells. Cells were cultured in medium containing bleomycin for 72 hours and then transferred to fresh FBS-free medium for 24 hours. SA-β-Gal staining and western blotting were then performed. As shown in Figure 4, the intensity of positive SA-β-Gal staining decreased when PTEN was overexpressed (Figure 4A, 4B), whereas the intensity of positive SA-β-Gal staining increased after PTEN was knocked down (Figure 4H–4I). The Akt pathway was activated after PTEN knockdown, and the expression levels of the aging-related marker, P21^ink4a^, were substantially higher in the knockdown cells than in the control cells (Figure 4J–4N). Up-regulation of PTEN caused Akt inactivation in senescence cells (Figure 4C–4G). These results supported our hypothesis that the loss of PTEN accelerates the senescence of AECs via activation of the Akt pathway.

### Knockdown of Akt2, but not Akt1, rescues cell from bleomycin-induced cell senescence

**Figure 5:**
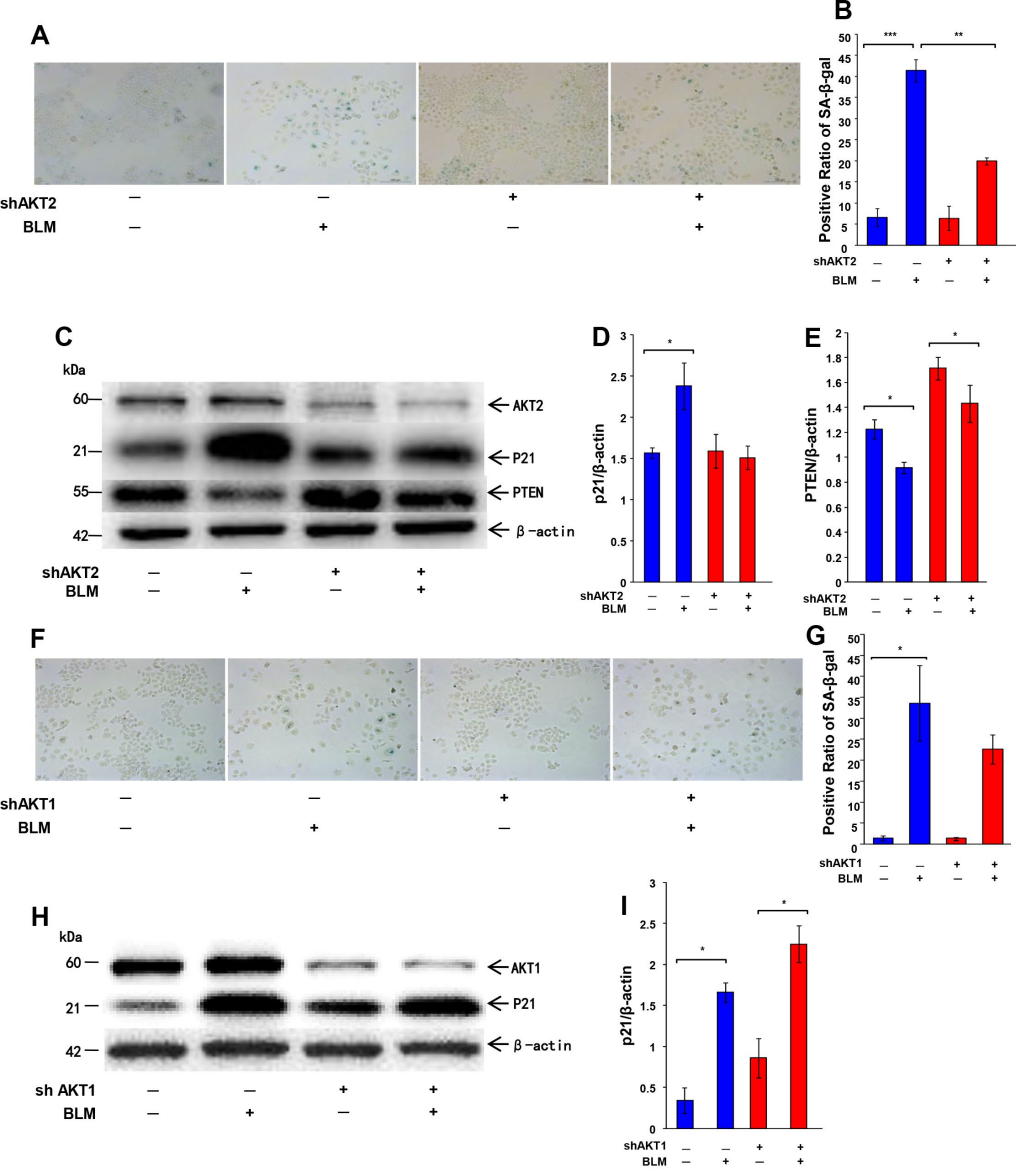
**Knockdown AKT2, but not AKT1, rescues cell from bleomycin-induced cell senescence.** The expression of AKT2 or AKT1 was knocked down by lentiviral vector in A549 cells followed by bleomycin (10 μg/ml) stimulation for 72 hours and transfer to fresh medium for 24 hours. (**A**, **B**) Cellular senescence was detected by SA-β-Gal staining (original magnification, 200×). (**C**–**E**) Western blotting was performed to confirm the change of P21^WAF1^, PTEN and AKT2. (**F**–**G**) Cellular senescence after AKT1 knockdown was analyzed by SA-β-Gal staining (original magnification, 200×). (**H**, **I**). Expression of P21^WAF1^ and AKT1 was confirmed by western blotting. Data are shown as the mean ± SEM, n ≥ 3 per group. *p < 0.05, **p < 0.01, ***p < 0.001. One-way ANOVA followed by Dunnett’s Multiple Comparison Test.

There are three members in the Akt kinase family, namely, Akt1, Akt2, and Akt3, which share a high degree of homology. Akt1 and Akt2 are broadly expressed in many tissues and cell types, whereas Akt3 is predominantly expressed in brain tissue [27, 28]. Each of the Akt isoforms has a distinct but overlapping function in the regulation of cell proliferation, cell apoptosis, protein synthesis, and the cell cycle [29]. Numerous clinical studies have indicated differential expression of Akt isoforms in different types of cancer and cells [30–33]. However, the consequences of suppression of Akt activation by PTEN in AEC senescence and its potential prevention of lung fibrosis are still unknown. Furthermore, it remains unclear if Akt1 and Akt2 both play comparable roles in AEC senescence. To examine if Akt and, particularly, which Akt isoform (Akt1 or Akt2) is involved in the development of AEC senescence, we used lentivirus to knockdown Akt1 or Akt2 in A549 cells to compare the senescence level in bleomycin-stimulated cells. Western blot analysis was used to verify Akt1 and Akt2 silencing. As shown in Figure 5C, 5H, both shRNAs targeting Akt1 and Akt2 (shRNA1 and shRNA2) decreased Akt1 and Akt2 protein levels. SA-β-Gal staining demonstrated that silencing of Akt1 had only a limited impact on cell senescence (Figure 5F, 5G). However, silencing of Akt2 significantly reduced cell senescence rates in bleomycin-stimulated AECs (Figure 5A, 5B) compared to the corresponding control shRNA. A similar result was found with measurement of the expression of the senescence-related marker, P21^WAF1^ (Figure 5C–5E, 5H–5I). This result indicated that only Akt2 is involved in bleomycin-induced AEC senescence.

### Inhibition of Akt activation attenuates AEC senescence

**Figure 6:**
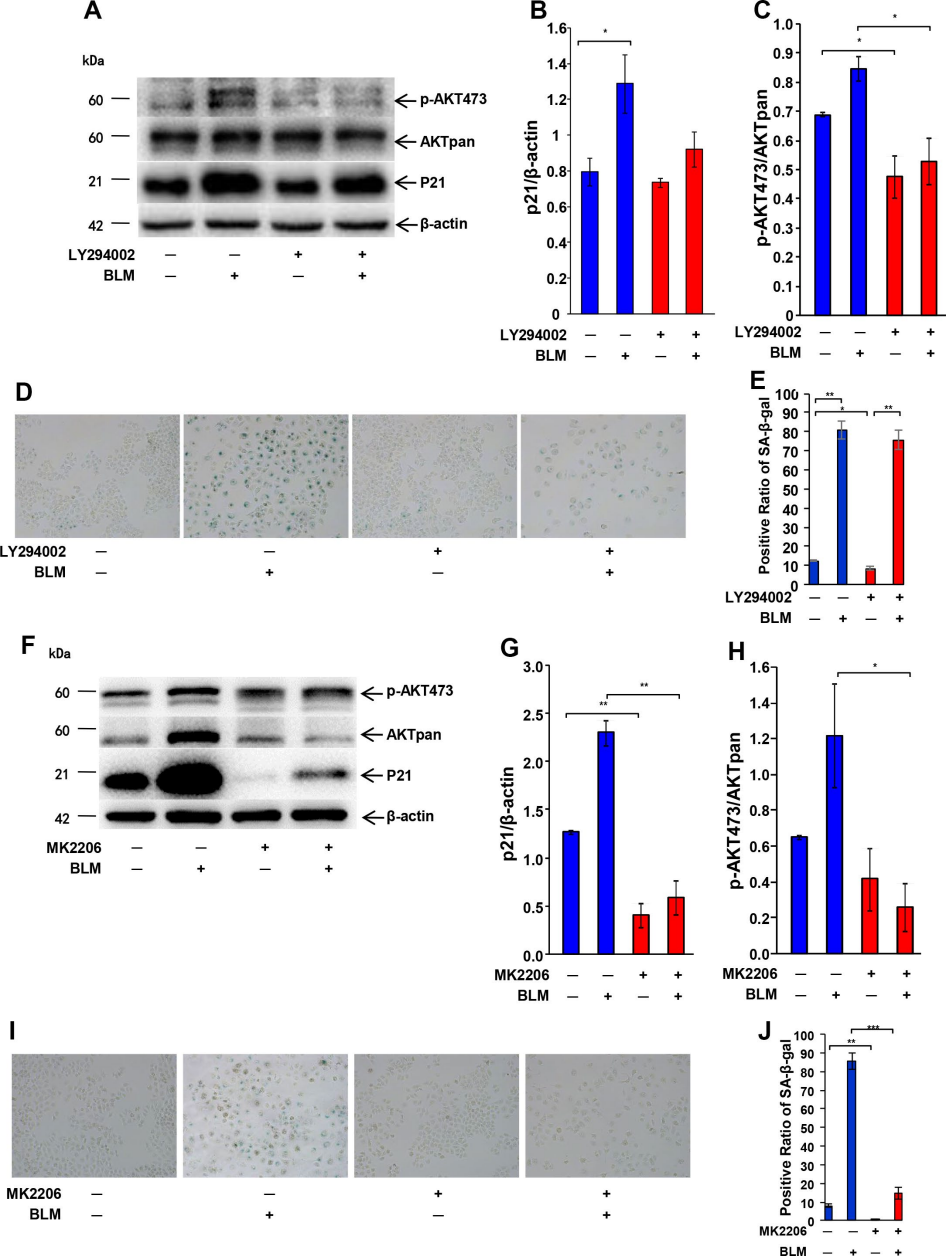
**Inhibition of Akt activation attenuates AEC senescence.** Inhibitor (20 μm/L LY294002 or 5 μg/ml MK2206) of the AKT pathway was added 1 hour before bleomycin (10 μg/ml). After 72 hours, the medium was changed to fresh medium for 24 hours. AKT inactivation and P21^WAF1^ expression induced by LY294002 (**A**–**C**) or MK2206 (**F**–**H**) were detected by western blotting. Cellular senescence after inhibitor LY294002 (**D**, **E**) or MK2206 (**I, J**) pretreatment was analyzed by SA-β-Gal staining. Data are shown as the mean ± SEM, n ≥ 3 per group. *p < 0.05. One-way ANOVA followed by Dunnett’s Multiple Comparison Test.

Because the activation of Akt is thought to accelerate cellular senescence, we observed the activation of Akt in senescent AECs, and we then verified if activated Akt also has a regulatory effect on AEC senescence. Two specific inhibitors of the Akt pathway were used. As shown in Figure 6, SA-β-Gal staining and the P21^WAF1^ senescence marker were significantly reduced after inhibition of Akt activation using LY294002 or MK2206 in bleomycin-stimulated AECs.

### Akt inactivation rescues AEC senescence in vitro

**Figure 7:**
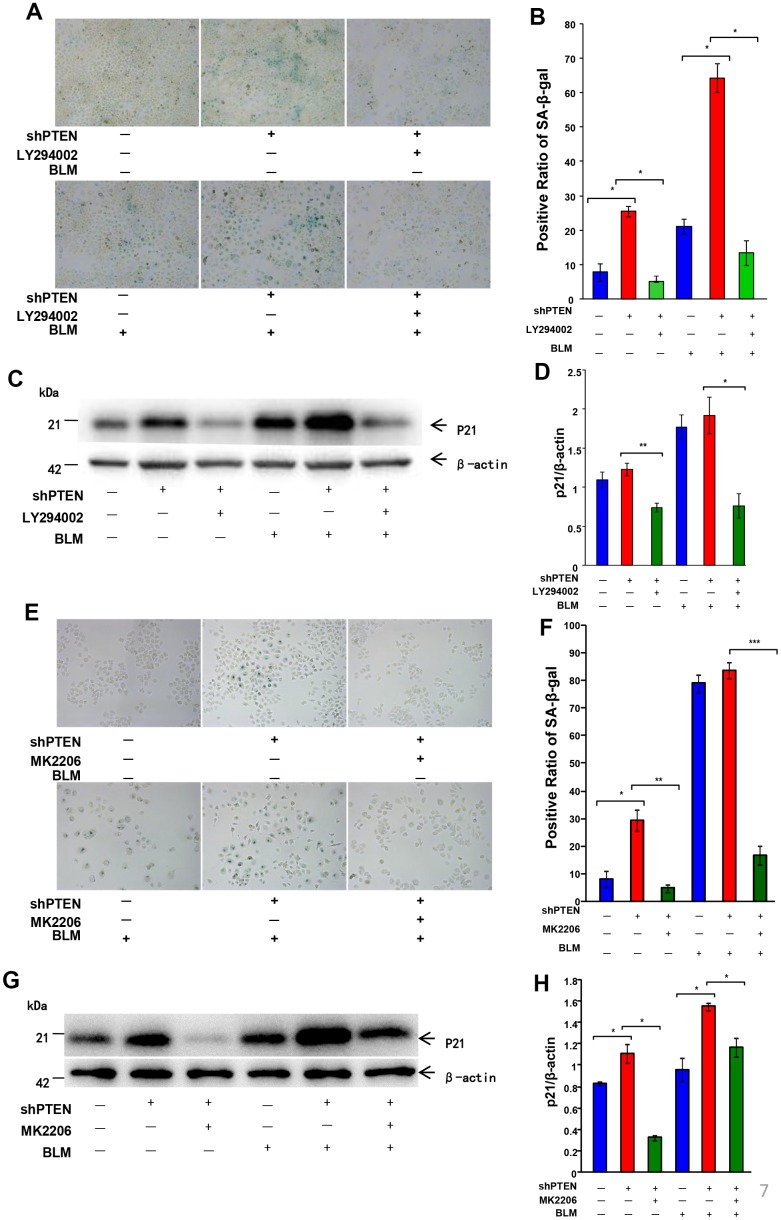
**Akt inactivation rescues AEC senescence in vitro.** After PTEN was stably knocked down, an inhibitor of the AKT pathway (20 μm/L LY294002 or 5 μg/ml MK2206) was added 1 hour before bleomycin (10 μg/ml) to A549 cells for 72 hours followed by a fresh medium transfer for 24 hours. (**A**, **B**) Rescue of cell senescence by LY294002 was determined by SA-β-Gal staining (original magnification, 200×), and decreased expression of P21^WAF1^ (**C**, **D**) was confirmed by western blot. Rescue of cell senescence by MK2206 was determined by SA-β-Gal staining (**E**, **F**) and P21^WAF1^ expression (**G**, **H**). Data are shown as the mean ± SEM, n ≥ 3 per group. *p < 0.05, **p < 0.01. One-way ANOVA followed by Dunnett’s Multiple Comparison Test.

We next explored if PTEN loss-induced AEC senescence depends on Akt activation. After stable knockdown PTEN, A549 cells were treated with LY294002 or MK2206 for 72 hours, and cells were then cultured in FBS-free medium for another 24 hours. As shown in Figure 7, PTEN loss induced senescence in A549, while inhibition of Akt by LY294002 or MK2206 rescues senescence. Both increased positive SA-β-Gal staining and P21^WAF1^ expression were decreased when Akt activation was blocked by LY294002 or MK2206 in A549 cells (Figure 7). To further confirm this finding, bleomycin was used to stimulate cells. PTEN-silenced A549 cells were pretreated with LY294002 or MK2206 for 1 hour, and bleomycin was then used to induce cell senescence for 72 hours. Cells were then cultured in fresh medium for an additional 24 hours. SA-β-Gal staining and western blotting showed that LY294002- or MK2206-pretreated cells presented lower intensity of positive SA-β-Gal staining and P21^WAF1^ protein expression. These results demonstrated that PTEN loss-induced AEC senescence depends on Akt activation.

### Akt inactivation attenuates senescence-related markers and pulmonary fibrosis in vivo

**Figure 8:**
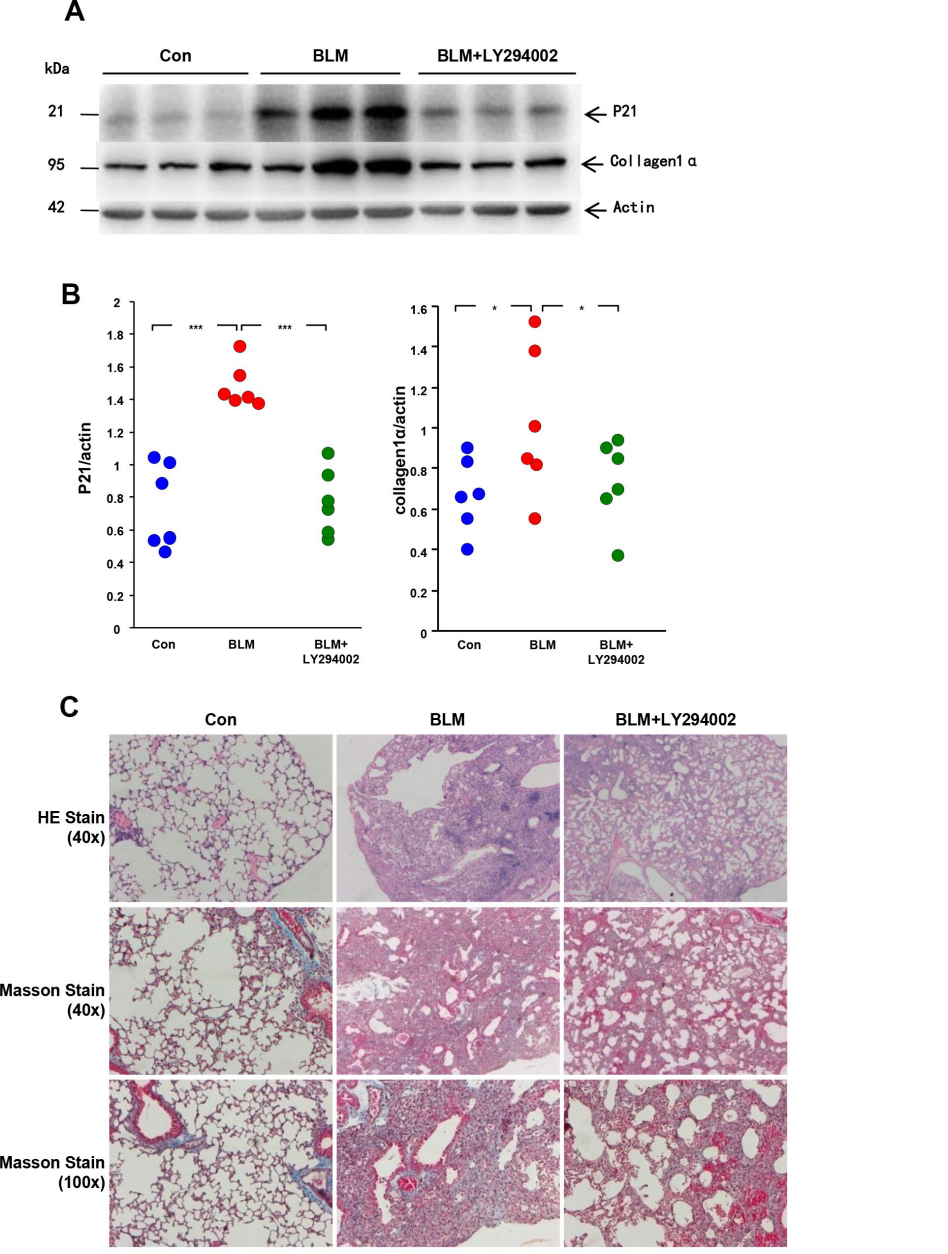
**Akt inactivation attenuates AEC senescence and pulmonary fibrosis in vivo.** C57BL/6 male mice were randomly divided into vehicle group (n = 6), bleomycin group (n = 6) and inhibitor group (n=6). Mice in the vehicle group were intratracheally injected with 50 μl of 0.9% saline, and mice in the bleomycin group and inhibitor group were injected intratracheally with 50 μl of 5 mg/kg bleomycin. From the day of bleomycin injection, mice in the inhibitor group were treated daily with LY294002 (50 mg/kg) through intraperitoneal injection. On day 14 after bleomycin or saline treatment, lungs were harvested. (**A**, **B**) Expression of the P21^WAF1^ senescent marker and the collagen1α fibrotic marker was detected by western blot analysis. Dots in the graph represent values for each individual mouse. *p < 0.05, ***p < 0.001. One-way ANOVA followed by Dunnett’s Multiple Comparison Test. (**C**) Representative results of HE and Masson staining of mouse lung tissues.

The bleomycin-induced mice pulmonary fibrosis model is a widely used animal model for pulmonary fibrosis. After exposure to bleomycin for 14 days, the expression levels of the senescence-related marker, P21^WAF1^, was significantly higher in mouse lungs, indicating that cellular senescence occurred in mouse lung tissue and that pulmonary fibrosis developed (Figure 8). Starting on the day of intratracheal injection with bleomycin (5 mg/kg in 100 μl saline), mice were intraperitoneally injected daily with LY294002 (50 mg/kg) for 14 days. Lungs were harvested for western blot analysis. LY294002 attenuated the P21^WAF1^ senescence marker in mouse lungs, resulting in significantly lower pulmonary fibrosis as assessed by lower collagen1α, hematoxylin and eosin (HE) and Masson Trichrome staining compared to mice treated with vehicle (Figure 8).

### PTEN physically associates with Akt

**Figure 9:**
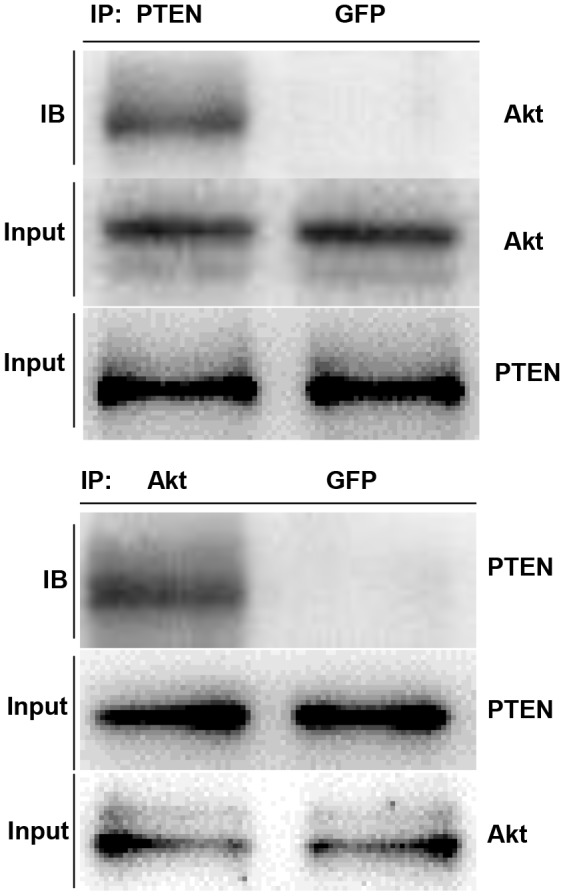
**PTEN physically associates with Akt.** In A549 cells, immunoprecipitation of PTEN resulted in the co-IP of AKT, and immunoprecipitation of AKT also resulted in the co-IP of PTEN.

To further investigate the interaction between PTEN and Akt, we performed a co-immunoprecipitation (co-IP) assay. As shown in Figure 9, immunoprecipitation of PTEN resulted in the co-IP of Akt in A549 cells. Immunoprecipitation of Akt resulted in the co-IP of PTEN. These results demonstrated that PTEN physically associates with Akt in A549 cells.

## DISCUSSION

Because increasing evidence has shown that IPF is an aging-related disease, great effort has been made to identify novel anti-senescence compounds for IPF therapy. Most studies regarding senescence in IPF are mainly focused on fibroblasts, and our recent study demonstrated that lung epithelial cells also play an important role in IPF by triggering collagen deposition in fibroblasts [34]. The present study further investigated the loss of PTEN participation in the pathogenesis of pulmonary fibrosis through regulating the senescence of AECs. We confirmed that PTEN loss-induced AEC senescence is AKT2 dependent.

Both genetic and epigenetic losses of the PTEN tumor suppressor gene are frequently observed in human cancers. Interestingly, while monoallelic loss or mutation of PTEN drives cellular proliferation, complete inactivation of PTEN induces a senescence response, termed PTEN loss-induced cellular senescence (PICS) that opposes tumor development [19]. Low levels of PTEN expression in fibroblasts and epithelial cells from IPF lungs have been detected. Mice with a constitutive deficiency of PTEN develop spontaneous fibrosis via intrinsic defects in fibroblasts and negative regulation of their proliferative responses [35]. A better understanding of the molecular mechanism that loss of PTEN drives senescence in IPF is of great relevance for the clinical development of anti-senescence treatments for IPF patients. However, the mechanism behind PTEN loss in AEC senescence remains elusive thus far.

To address this question, we used stable PTEN-overexpressing and PTEN knockdown cells to establish senescence cell models. We observed increased Akt activation and deteriorated senescence in the PTEN knockdown cell models. However, decreased Akt pathway activation and attenuated senescence were present in the PTEN-overexpressing cell models. These results indicated that the Akt pathway is an important potential barrier to PTEN loss-driven cell senescence.

Akt activation is strongly associated with regulating survival and differentiation of myofibroblasts in the setting of pulmonary fibrosis as it is known to regulate many fibrotic remodeling processes in IPF [36]. Akt signaling has been reported to play a central role in controlling fibroblast proliferation and survival [37–40]. However, the precise role of the Akt pathway in AEC senescence remains unknown.

According to previous studies, Akt1-mediated mitophages contribute to alveolar macrophage apoptosis resistance, which is required for pulmonary fibrosis development [31]. Akt1−/−mice are protected from chronic hypoxia-induced pulmonary vascular and tissue remodeling [41]. The efforts to dissect the molecular mechanisms of Akt isoform-specific signaling will provide new insights for designing more effective and selective therapeutics for IPF treatment. In mammals, the Akt family consists of three isoforms, namely, Akt1, Akt2 and Akt3. Because Akt3 is predominantly expressed in brain tissue, we only studied the Akt1 and Akt2 isoforms. Our study showed that inhibition of Akt pathway activation or knockdown of Akt2 rescues bleomycin-induced cell senescence. Our animal study further demonstrated that inhibition of Akt activation attenuates pulmonary fibrosis. These results were supported by a recent study showing that inhibition of Akt phosphorylation in lungs improves lung function in asthma and fibrosis in mice [42]. Akt2-deficient mice are protected against bleomycin-induced pulmonary fibrosis and inflammation [43].

While fibroblasts were not found to be affected to a large extent in IPF, recent studies have shown that epithelial cells represent a major cell type that is affected by senescence [44]. Senescent epithelial cells secrete several mediators in the SASP, which have been shown to directly influence their surrounding microenvironment, thereby triggering IPF [45]. Studies have revealed that AEC injury is sufficient to initiate lung fibrosis [12, 13]. Senolytic treatment targeting fibrotic lung epithelial cells in vitro and ex vivo attenuates pulmonary fibrosis [15]. These findings indicate that AEC senescence is a promising target for developing novel IPF therapy. The present study also suggested that the senescence of AECs may be a profibrotic initiator in IPF and that the PTEN/Akt pathway may be a new candidate for senolytic drugs, which may advance treatment of IPF.

In conclusion, the present study confirmed that AECs accelerate cellular senescence in IPF. We further showed that PTEN loss-induced AEC senescence occurs in an Akt-dependent manner, which may help identify a potential target for anti-senescent therapy in treatment of lung fibrosis.

## MATERIALS AND METHODS

### Patients and specimens

The present study was approved by the Ethics Committee of the Medical School of Nanjing University. Written informed consent was obtained from all the patients enrolled in this study. All human lung tissues of IPF (n = 12) were obtained from the Department of Lung Transplantation, Wuxi People’s Hospital, Wuxi, China. Normal peripheral tissues (n = 12), where were used as controls, were obtained from patients who underwent lobectomy in the Thoracic Surgery Department of Nanjing Drum Tower Hospital of the Affiliated Hospital of Nanjing University Medical School. All diagnoses of IPF were made in accordance with the ATS/ERS criteria for IPF 2011. For the 12 patients who provided normal lung tissues, the average age was 63.16 ± 4.62 years, and 1 female and 11 males were included. For the 12 IPF patients, the average age was 61.83 ± 5.49 years, and 1 female and 11 males were included.

### Cell culture

A549 cells were purchased from American Type Culture Collection (Rockville, MD, USA). Cells were cultured in DMEM (Gibco, Australia) supplemented with 10% FBS (Gibco) and 1X penicillin–streptomycin solution (Thermo Fisher Scientific, USA) at 37°C in 5% CO2.

### Reagents

Bleomycin was purchased from Nippon Kayaku Co Ltd. (Japan), dissolved in 0.9% saline and stored at −20°C. LY294002, an inhibitor of PI3K/AKT, was purchased from Sigma-Aldrich (USA), dissolved in DMSO (Sigma, USA) and stored at −20°C.Diluted to working concentration of 20μM/L for cell culture. MK2206, an Akt pathway inhibitor, was purchased from Selleckchem, dissolved in DMSO (Sigma, USA) and stored at −20°C. Diluted to 5μg/ml when applied to cells.

### Mouse pulmonary fibrosis models

Six- to eight-week old male SPF C57BL/6 mice (Shanghai Laboratory Animal Center, Chinese Academy of Sciences, Shanghai, China) were randomly divided into the vehicle group (n = 6), bleomycin group (n = 6) or inhibitor group (n=6). All protocols were approved by the Ethics Committee for Animal Research of Medical School of Nanjing University. Mice in the vehicle group were intratracheally injected with 50 μl of 0.9% saline, and mice in the bleomycin and inhibitor groups were injected intratracheally with 50 μl of 5 mg/kg bleomycin. From the day of bleomycin injection, mice in the inhibitor group were treated daily with LY294002 (50 mg/kg) through intraperitoneal injection. On day 14 after bleomycin or saline treatment, mice were sacrificed, and lungs were collected for subsequent experiments.

### Cellular aging model

A549 cells were stimulated with specific concentrations of bleomycin for 72 hours followed by culture in FBS-free medium for 24 hours to generate the cellular senescence model. Aging-related markers, including p21 and senescence-associated β-galactosidase (SA-β-Gal), were then tested to confirm the senescent state of cells.

### Lentiviral transfection

To generate stable genetic knockdown of PTEN and Akt, lentiviruses were used as a vector to carry the interference sequence. Vectors loaded with the targeting gene or controls were constructed by GenChem (Shanghai, China). Lentivirus was added to 1 ml of complete medium per well of a 6-well slide in addition to 5 μg/ml polybrene. After 8 to 12 hours, the medium was replaced with fresh complete medium without lentivirus and polybrene for another 72 hours. Green fluorescence was observed for rough estimates of transfection efficiency, and total cellular protein was extracted to further confirm the transfection efficiency by western blotting. The sequence of edited genes are as follows: Table 1.

**Table 1:** The sequence of edited genes.

**Knocked down gene**	**Target seq**	**Oligo**				
		**5′**	**STEM**	**Loop**	**STEM**	**3′**
PTEN	GACGAACTGGTGTAATGATAT	Ccgg	GACGAACTGGTGTAATGATAT	CTCGAG	ATATCATTACACCAGTTCGTC	TTTTTg
		aattcaaaaa	GACGAACTGGTGTAATGATAT	CTCGAG	ATATCATTACACCAGTTCGTC	
Akt1	gaGGCCAAGTCCTTGCTTTCA	Ccgg	gaGGCCAAGTCCTTGCTTTCA	TTCAAGAGA	TGAAAGCAAGGACTTGGCCTC	TTTTTg
		aattcaaaaa	gaGGCCAAGTCCTTGCTTTCA	TCTCTTGAA	TGAAAGCAAGGACTTGGCCTC	
Akt2	AAGGTACTTCGATGATGAA	Ccgg	acAAGGTACTTCGATGATGAA	CTCGAG	TTCATCATCGAAGTACCTTGT	TTTTTg
		aattcaaaaa	acAAGGTACTTCGATGATGAA	CTCGAG	TTCATCATCGAAGTACCTTGT	

The vector used for upPTEN was constructed by GenChem (Ubi-MCS-3FLAG-SV40-EGFP-IRES-puromycin). Empty vectors were applied as controls.

### Senescence-associated β-galactosidase staining

SA-β-Gal staining was performed according to the manufacturer’s instructions. The Senescence β-Galactosidase Staining Kit was purchased from Beyotime Biotechnology (Shanghai, China). Cells on 6-well chamber slides were fixed with 4% formaldehyde for 10 min at room temperature, rinsed three times with PBS for 5 min and incubated with freshly prepared SA-β-Gal staining solution at 37°C overnight. Slides were washed twice with PBS for 10 min at room temperature. Images were captured using a microscope equipped with a digital camera (Eclipsee800, Nikon). For each slide, at least three fields were obtained to calculate the SA-β-gal intensity.

### Western blot

Whole protein from cell lysates or lung tissues was extracted according to manufacturer’s instructions (Keygene, China). Equal amounts of protein were separated on 10% SDS-PAGE gels, blotted onto PVDF membranes (Merck Millipore, Germany), blocked with 5% skim milk in TBST and incubated with primary antibody at room temperature for 4 hours or overnight. Membranes were incubated with HRP-conjugated secondary antibody for one hour, and protein expression was detected using ECL (Merck Millipore, Germany). Primary antibodies against P21, PTEN and Akt were purchased from Cell Signaling Technology (USA).

### Immunohistochemical staining

Tissues were fixed in formalin and embedded in paraffin. Immunohistochemical staining for target proteins and hematoxylin–eosin staining (HE) were performed. Four-micron-thick sections were deparaffinized in xylene and rehydrated in graded alcohol. Endogenous peroxidase was quenched with 3% aqueous hydrogen peroxide for 15 min. Antigen retrieval was then performed in a pressure cooker. Sections were incubated with 10% normal goat serum to block any nonspecific reaction. After primary antibody incubation overnight at 4°C, horseradish peroxidase-conjugated secondary antibody was added for 20 min, and 3,3-diaminobenzidine tetrahydrochloride (DAB) was added for 10 min at room temperature. Finally, sections were dehydrated and mounted.

### Immunofluorescence assay

Fresh lung tissues were fixed in 4% paraformaldehyde, embedded in paraffin and cut into 4-μm sections. After deparaffinating, rehydration and retrieval, tissues were incubated with primary antibodies overnight at 4°C. After rinsing several times with PBS, sections were incubated with Alexa-488- or Alexa-568-conjugated secondary antibodies (Invitrogen, United Kingdom) for 2 hours. Finally, DAPI was added onto slides for 10 minutes. Images were obtained with a ZEISS Imager A1 fluorescence microscope (Gottingen, Germany). Immunofluorescence was performed using the following primary antibodies: rabbit monoclonal anti-P21 and anti-PTEN antibodies (Abcam, United Kingdom); and mouse monoclonal anti-SPC antibody (Santa Cruz Biotechnology, CA, USA).

### Masson’s trichrome staining

Lung tissues were fixed in 4% paraformaldehyde at room temperature overnight followed by dehydration with gradient alcohol and embedding in paraffin. Sections of 5 μm were subjected to Masson’s trichrome staining according to the manufacturer’s instructions (Solarbio, China).

### Co-immunoprecipitation

Cells were lysed in buffer containing 50 mM Tris-HCl (pH 7.4), 150 mM NaCl,1% NP-40 and protease inhibitor. Cell lysates were placed on ice for 1 hour with mixing every 10 minutes and then centrifuged at 13000 g at 4 °C for 20 minutes. Protein was first incubated with the following primary antibodies at 4 °C for 1 hour: mouse monoclonal anti-PTEN, anti-AKT or anti-GFP (control antibody) antibodies (Santa Cruz Biotechnology, CA, USA). Protein A/G plus-Agarose (20 μl; Santa Cruz Biotechnology, CA, USA) was then added at 4 °C for 4 hours. After washing 4 times with PBS, samples were resuspended in 40 μl of electrophoresis sample buffer and heated at 70 °C for 10 minutes. The supernatant was subjected to SDS-PAGE and western blotting.

### Statistical analysis

Data were expressed as the mean values ± SEM. Student’s t-test (2-tailed) was used to determine differences for experiments between two groups, and one-way ANOVA was applied to analyze differences for experiments with more than two groups. *p* value less than 0.05 was accepted as statistically significant.
